# The influence of late retirement on health outcomes among older adults in the policy context of delayed retirement initiative: an empirical attempt of clarifying identification bias

**DOI:** 10.1186/s13690-021-00582-8

**Published:** 2021-04-26

**Authors:** Jiannan Li, Bocong Yuan, Junbang Lan

**Affiliations:** 1 Institute of Advanced Studies in Humanities and Social Sciences, Beijing Normal University, Zhuhai, 519087 China; 2 School of Tourism Management, Sun Yat-sen University, West Xingang Rd. 135, Guangzhou, 510275 China

**Keywords:** Delayed retirement, Late retirement, Difficulty in physical functioning, Problems of cognitive status, Older adults

## Abstract

**Background:**

The deepening population aging is urging policy makers to launch delayed retirement initiative, when the society is faced with unprecedented challenges of shrinking labor supply, heavier pension burdens and slowing economic growth. However, the health outcomes of late retirees receive scarce attention due to the intrinsic identification difficulties (i.e., (1) self-selection bias – older adults with predetermined ill-health are less likely to delay retirement. (2) there can be situations where the status of late retirement has terminated at the time of interview, although he/she has ever delayed retirement). To fill in this research gap, this study examines the effect of late retirement on the difficulty in physical functioning and problems of cognitive status among older adults.

**Method:**

Using the data from China Health and Retirement Longitudinal Study (CHARLS-2015 harmonized, and CHARLS-2018), this study investigates the influence of late retirement (year 2015) on the difficulty in physical functioning and problems of cognitive status (year 2018) among older adults. A series of robustness checks are also conducted.

**Results:**

Empirical results show that late retirement is associated with better physical functioning and cognitive status. The influence remains robust after considering potential self-selection bias and the sensitivity of including/excluding older adults who have past late retirement experience but have no longer been late retirees at the time of survey.

**Conclusion:**

This study suggests that older adults might benefit from the engagement in late careers in their physical and cognitive functioning.

## Background

Population aging has increasingly become an urgent global issue. Economic challenges emerge as the social dependency ratio keeps growing [1], which manifests as a potential shortage of workforce in the labor market and a surge in costs related to pensions and health care [2]. Population aging presents a challenge to the future viability of pension systems insofar as there will be many more retirees but fewer active workforce to support the continual operation of pension systems over a long period of time in future [3]. In order to offset the undesired fiscal implications for pension systems, many governments around the world call for the deferment of legal retirement age with the aim of delaying the exit of workforce from the labor market [4]. Legislative efforts further speed up this process. In the United States, for example, an amendment of Age Discrimination in Employment Act (ADEA) has eliminated mandatory retirement, which at least has changed the social perception of “normal” retirement age [5], and Americans work for longer period of time than earlier birth cohort to claim social security benefits [6]. Policymakers engage in undoing the remaining disincentives to private and public pensions or resolving information-related labor market imperfections to encourage working at an old age [7]. And these practices serve a variety of social goals, including to counteract the slowdown in the growth of labor force and to shore up the finances of social security and medical insurance [7]. In developing countries like China, the life expectancy makes great progress and the legal retirement age stipulated decades ago is considered to lag behind social development. The gender specific legal retirement age in China (60 years-old for males and 55 years-old for females) is not just inconsistent with the fact that the life expectancy is longer for females than males [8], but also is criticized for generating potential gender disparity in career development ceiling.

Although great research attention is paid to the fiscal impact of delayed retirement for governments, much less has been paid to the health status of late retirees. As a matter of fact, the occurrence of events such as retirement or sudden reduction of social participation may also have a great impact on the physical health and cognitive status of older adults [9]. However, current research can just provide a few evidences showing that early exit of the labor market can negatively affect health status, or examining the impact of retirement on the health of older adults. Direct empirical evidence about the impact of “*late retirement”* on health outcomes of older adults remains quite insufficient. For example, it is shown that early exit of the labor market can increase all-cause mortality rate by 2.4% for blue-collar workers [10], whereas the modest extension of retirement age can reduce the all-cause mortality rate of old workers [11]. In addition, it is found that late retirement reduced the risk of depression by 5.5% in men and 6.4% in women aged 62–65 [11]. About 50.8% of adults who exit labor market early can experience the decline in health conditions, such as depression, physical illness characterized with pain in lower extremities, shortness of breath, limited mobility, and leg pain when walking [12]. However, it is still not appropriate to simply infer the effect of late retirement on health of older adults from the results of the effect of retirement or early exit of labor market on health status.

This study intends to enrich the research on associations of late retirement and health status among older adults. Firstly, health outcomes of late retirement have not received enough discussions, although population aging has been a global concern with bringing about a series of problems for many countries. Prior studies have noticed that the delayed retirement policy can influence the labor supply of labor market and the sustainability of pension system. However, rare research focuses on the health status among late retirees. This study thus tries to fill in the research gap by exploring the impact of late retirement on physical health status and cognitive status among older workers. Secondly, although there some attention paid to the impact of retirement on health outcomes of older adults, rare effort has been paid to the health implications of late retirement. The health outcomes of late retirement may not be obtained hands-down through simple inference from that of retirement. This study is among the earliest attempt to explore this under-investigated issue. Thirdly, this study can also enrich relevant research regarding the health of older workers. Previous studies focus on whether older workers’ working ability and efficiency could be susceptible to their age, and do not meticulously differentiate older workers into groups before and after the legal retirement age. In turn, this study specifically focuses on late retirees, and extends the research focus to the influence of the late retirement status on their health.

## Literature review

### The background of delayed retirement initiative proposed by governments

Population aging is one of the greatest social and economic challenges for many countries around the world. In Europe, the ratio of the population over 65 in age to age between 18 and 65 is expected to increase from 25% at present to about 50% in 2060 [13]. The trend of population aging and early unemployment in OECD countries leads to claims that future pension costs will greatly increase and become unsustainable [14]. Therefore, governments around the world actively seek ways to encourage late careers of older workers [15]. The policy of delayed retirement emerges in the background of this era. The primary governmental goal built in delayed retirement policy is to limit generosity or welfare benefits of early retirement plans, so that people can be encouraged to extend their careers to relieve fiscal pressures [16].

The problem of population aging requires policymakers to find effective incentives to encourage older adults to stay in the labor market and work longer [17]. Some of countries hope to promote longer working lives by reducing incentives for early exit and rewarding continued work [18]. The factors that drive older adults to choose postponing retirement include the extension of full retirement age of social security, the change from fixed income pension to defined contribution pension, and the lower physical demands of work [19]. In addition, lifelong continuing education and training measures can increase the qualification of older workers, and thereby increase their potential employability and their willingness to work longer hours [20]. Netherlands has encouraged delay retirement since the 1990s. Family-based bonus pension plans were phased out in the mid-1990s. In 2009, Netherlands introduced a new policy allowing older people an annual bonus after 62 years old, according to their age, birth group, and income [11]. In Germany, one-year early retirement will result in a 3.6% reduction in pension, whereas one-year delayed retirement will lead to a 6% increase in pension [21]. In Germany, the pension reform has decreased the internal rate of return of early retirees from 2.4 to 1.2% for males and from 5.2 to 3.7% for females [22].

China has also increasing paid attention to this issue. Recent studies show that the current retirement policy in China no longer meets the balance between heavy pension burden and economic development due to the aggravation of population aging caused by lower birth rate [21]. China tries to adopt appropriate delayed retirement rewards and early retirement fines to encourage elderly workers to make rational decisions about delayed retirement [23]. In 2016, the State Council of China approved a plan that gradual delay in retirement age will be set in the next 5 years. Previously, some scholars believed that the flexible retirement policy should be implemented, based on specific industrial characteristics, physical condition, education, and other factors [24]. To sum up, the delayed retirement policies around the world are motivated mainly around the pension systems.

### Participation in late career and physical functioning among older adults

Extended life expectancy makes the participation in late careers feasible [6]. The career participation in late-life may benefit individual health [25], as physical activity maintenance throughout modest work engagement in late life is associated with lower incidence of many chronic symptoms [26], and can lower down the mortality risk of retirees who are not in healthy condition [27]. Among people aged 60 years old and above, productive engagement in work such as paid work, voluntary work and care-giving is shown associated with less physical disability, better cognitive status, and self-rated health [28]. Prior studies show that retirees who are no longer working have a higher likelihood of suffering from two or more diseases than their counterparts who remain in the full-time labor market after 65 years old (45% vs. 27%) [29]. People who still engage in full-time work after their retirement age are reported to not experience an increase in physical dysfunction with age, but in contrast, their counterparts who withdraw from the labor market after retirement age are reported to have higher chances of mobility difficulties [29].

Maintenance of physical activity functioning can be realized through daily work for older adults who participate in full-time work [30]. Before retirement, work-related physical activities (e.g., physical labor and transportation) account for major part of daily physical activity engagement [31, 32]. A 13-year follow-up study found that retirees were more likely to spend no time in physical activities than working seniors, and the percentage of retirees who spent almost no time in physical activities at work dropped sharply, declining from 90 to 55% [33]. Active workers have increased their physical activities because of job demands, but retirees have not. In short, engagement in work in late life could increase opportunities of daily physical exercise for older adults [33], and provide support for the lifestyle, social relations and achievements that can improve physical functioning of older adults [34].

### Participation in late career and cognitive status among older adults

Retirement is an important transition in one’s life that one leaves usual work environment and returns home, and such change may affect cognitive status of older adults [35]. Although cognitive decline is common in older adults, the rate of decline is highly variable [36]. Cognitive decline of retirees is faster than that of those who continue working [37]. Prior studies show that older adults in employment outperform the retired especially in the aspects of computational ability and fluency [38]. After retirement, the motivation of cognitive rehabilitation activities is less, and thus the cognitive ability will decline faster [38]. According to a study based on a global assessment model, 60 years-old individuals can delay cognitive aging by 1.38 years by engagement in work, and by 1.75 years by engaging in regular charity activities, voluntary activities, or voluntary work [39].

Engagement in work brings people daily social activities that are related to lifelong cognitive ability [40], especially for older adults [41]. Therefore, when older adults reduce their daily social participation and interaction due to retirement, they will feel socially isolated, which is closely associated with the decline in cognitive ability [42]. Therefore, work participation is one of the effective means for older adults to prevent decline in cognitive ability, by giving frequent and high-intensity social activities, and providing unique opportunities to maintain their cognitive status [41]. In addition, some studies have shown that complex work has a positive impact on cognitive function, as a higher level of cognitive reserve can help prevent or slow down the process of aging-related neurodegeneration [43]. There can be a relation between the activity that needs to mobilize more cognitive resources and the alleviation of cognitive aging [44].

Social activities and social participation can delay the decline in cognitive ability of older adults [45]. Over time, older adults with higher social participation have lower levels of physical and cognitive limitation [46]. Socially integrated lifestyles have been shown beneficial to the alleviation of cognitive decline in later life [41]. Higher levels of social participation are associated with better cognitive status, lower levels of depression and other mental health problems in later life [47]. Social participation, such as work participation, family maintenance, social activities, and community services, can also slow down the decline in cognitive function [48]. Engagement in work in late life can provide individuals with social support [49] and self-efficacy [50]. Since involving allocating many cognitive resources for professional and daily life activities, engagement in work helps employees maintain a high level of cognitive function. These findings can support the bright side of delayed retirement regarding individual health, such as attention and memory [51].

## Method and materials

### Description of data

**Fig. 1:**
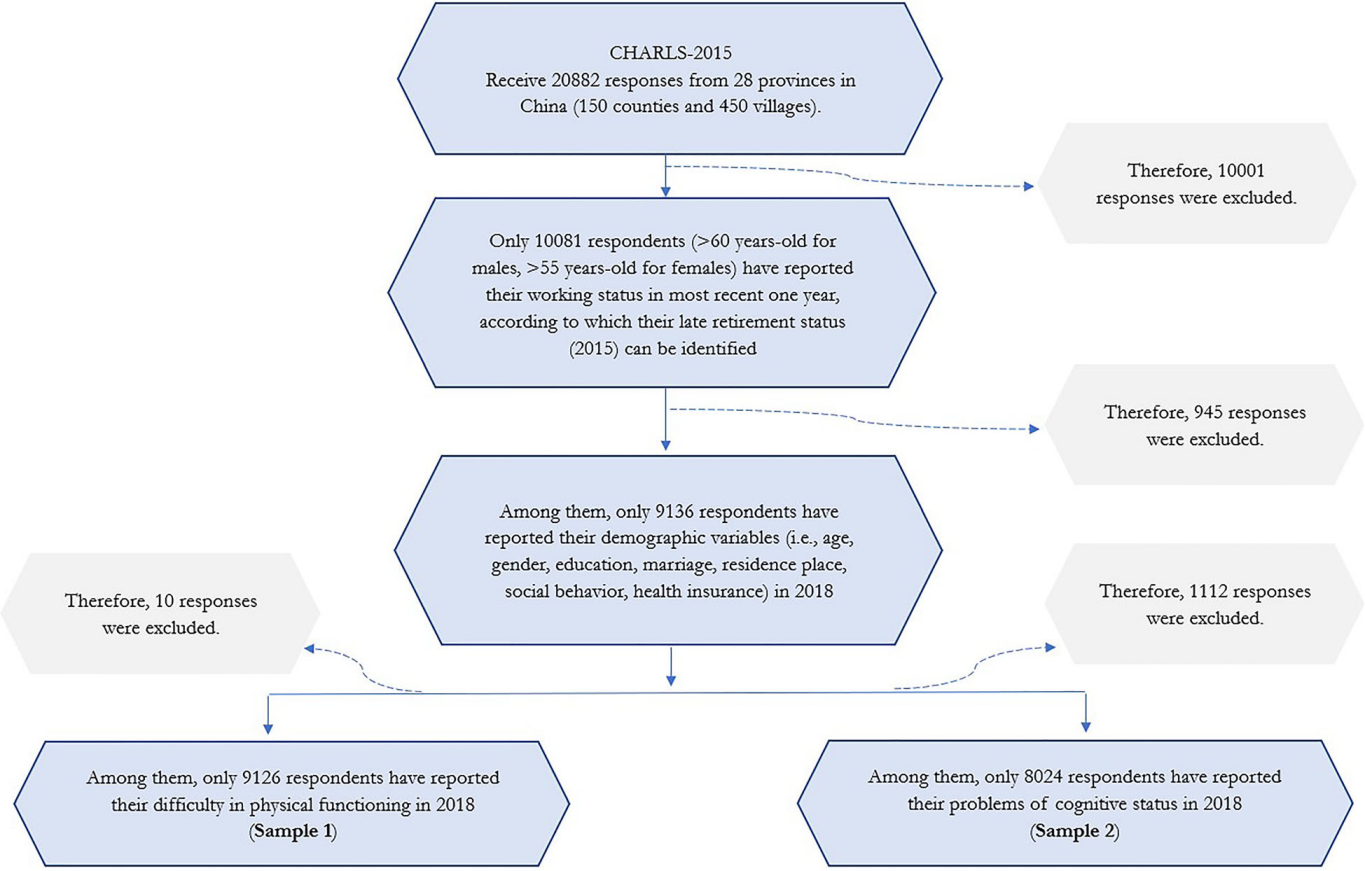
The flowchart of samples

The data from China Health and Retirement Longitudinal Study (CHARLS-2018 wave) and CHARLS-2015 (harmonized version) are combined and applied in this study. The survey of CHARLS is initiated with aiming at tracking health situation of old-aged adults, with using stratified random sampling. This survey covers 150 counties in China, with 52.6% respondents from rural areas and 47.4% from urban areas. This study is exempted from reviewing by institutional review board as this study applies publicly available data. More details about analytical sample are shown in the flowchart (see Fig. 1).

### Variables

*Difficulty in physical functioning (year 2018)* is measured by the 7-items scale used by CHARLS-2018. Questions are shown as “Do you have any difficulty with running or jogging about 1 km?”, “Do you have difficulty with getting up from a chair after sitting for a long period?”, “Do you have difficulty with climbing several flights of stairs without resting?”, etc. (coded 1 = No, I don’t have any difficulty...4 = I cannot do it). The mean score of items is used as the measure of the variable.

*Problems of cognitive status (year 2018)* are measured by the Informant Questionnaire on Cognitive Decline in the Elderly (IQCODE) scale [52]. Questions are shown as “How well at recognizing the face of family members and friends?”, “How well at recognizing the name of family members and friends?”, etc. (coded 1 = much better; 2 = improved; 3 = not much changed; 4 = gotten worse; 5 = much worse). The mean score of items serves as the measure of the variable.

*Late retirement (year 2015)* is an indicator variable for which a respondent is labeled as a late retiree (coded as 1) if he (or she) meets both of the following conditions that being engaged in work currently since the most recent year, and at the age of over 60 years old (or over 55 years), whereas a respondent is labeled as a non-late retiree (coded as 0) if he (or she) reports the status of being not work in the most recent year, and at the age of over 60 years old (or over 55 years). The cut-off point of age is gender-specific and according to the regulation of legal retirement age in China.

Besides, we also control a series of demographic variables such as gender (coded 1 = Male; 2 = Female), age (coded as the years of age), education (coded 1 = less than lower secondary; 2 = upper secondary & vocational training; 3 = tertiary), marriage status (coded as 0–1 binary variable for each type of marital status), residence place (coded as 0–1 binary variable for each type of residence place). Since health insurance could be associated with health status of older adults [53], this study also controls the effect of health insurance status (coded 1 = Participation in at least one of health insurance including Urban Employee Basic Medical Insurance (UEBMI), Urban Resident Basic Medical Insurance (URBMI), and New cooperative medical scheme (NCMS); 0 = otherwise). The effect of social behavior, which can be associated with the problems of cognitive status of older adults [54], is controlled as well (coded 1 = Engagement in one of social behaviors as listed in Table 1; 0 = otherwise). More details about variables have been shown in Table 1, and descriptive statistics of variables are shown in Table 2.

**Table 1:** Description of variables

Variables	Description
*Difficulty in physical functioning (of year 2018)*	In year 2018, the mean score of the following items. “Do you have any difficulty with running or jogging about 1 km?”, “Do you have difficulty with getting up from a chair after sitting for a long period?”, “Do you have difficulty with climbing several flights of stairs without resting?”, “Do you have difficulty with stooping, kneeling, or crouching?”, “Do you have difficulty with reaching or extending your arms above shoulder level? (he/she is regarded as not having difficulty only if he/she can extend both of his/her arms, otherwise he/she is regarded as having difficulty.)”, “Do you have difficulty with lifting or carrying weights over 10 jin, like a heavy bag of groceries?”, “Do you have difficulty with picking up a small coin from a table?”. (=1, No, I don’t have any difficulty... =4, I cannot do it).
*Problems of cognitive status (of year 2018)*	In year 2018, the mean score of the following items. “how well at recognizing the face of family members and friends?”, “how well at recognizing the name of family members and friends?”, “how well at remembering things about family and friends, such as occupations, birthdays, and addresses?”, “how well at remembering things that have happened recently?”, “how well at recalling conversations a few days later?”, “how is at forgetting what was about to say in the middle of conversations?”, “how is at remembering [his/her] address and telephone number?”, “how is at remembering what day and month it is?”, “how is at remembering where things are usually kept?”, “how is at remembering where to find things which have been put in a different place from usual?”. (=1, much better; =2, improved; =3, not much changed; =4, gotten worse; =5, much worse).
*Late retirement status of 2015*	In year 2015, the status of late retirement is coded 1, if he (or she) reports the status of working in the most recent year and he (or she) is aged > 60 (or > 55); And the status of late retirement is coded as 0, if he (or she) reports the status of not working in the most recent year and he (or she) is aged > 60 (or > 55). The cut-off point is gender-specific and according to the regulation of legal retirement age in China.
*Social behavior*	=1, if he/she has one of the following social behavior; =0, otherwise. “Interacted with friends”, “Played Ma-jong, played chess, played cards, or went to community club”, “Provided help to family, friends, or neighbors who do not live with you”, “Went to a sport, social, or other kind of club”, “Took part in a community-related organization”, “Done voluntary or charity work”, “Cared for a sick or disabled adult who does not live with you”, “Attended an educational or training course”, “Stock investment”, “Used the Internet”.
*Insurance*	=1, if he/she has at least one of the following health insurances, including Urban Employee Basic Medical Insurance (UEBMI), Urban Resident Basic Medical Insurance (URBMI), and New cooperative medical scheme (NCMS); =0, otherwise.
*Gender*	=1, Male; =2, Female.
*Age*	The age of respondents.
*Education*	=1, less than lower secondary; =2, upper secondary & vocational training; =3, tertiary.
*Marriage*	Indicator (0–1 binary) variable of the following respective status: married; partnered; separated; divorced; widowed; never married.
*Residence place*	Indicator (0–1 binary) variable of the following respective places: central city/town; urban-rural integration zone; rural; special zone.

**Table 2:** Descriptive statistics

Variables	Freq.	%
**Sample 1**		
Difficulty in physical functioning (2018)		
*Range [1, 2)*	6020	65.97
*Range [2, 3)*	2438	26.71
*Range* [3, 4]	668	7.32
Late retirement (2015)		
*No*	4682	51.30
*Yes*	4444	48.70
Social behavior		
*No*	4900	53.69
*Yes*	4226	46.31
Health insurance		
*Insurance beneficiary (Yes)*	8620	94.46
*Insurance beneficiary (No)*	506	5.54
Gender		
*1. male*	3763	41.23
*2. female*	5363	58.77
Age		
55–65	2694	29.52
66–75	4438	48.63
> 75	1994	21.85
Education		
*Less than lower secondary*	8420	92.33
*Upper secondary & vocational training*	579	6.35
*Tertiary*	120	1.32
Marriage		
*Married*	7109	77.91
*Partnered*	343	3.76
*Separated*	13	0.14
*Divorced*	37	0.41
*Widowed*	1568	17.18
*Never married*	55	0.60
Residence place		
*Central of City/Town*	1523	16.69
*Rural integration zone*	517	5.67
*Rural*	7060	77.36
*Special zone*	26	0.28
**Sample 2**		
Problems of cognitive status (2018)		
*Range [1, 2)*	39	0.49
*Range [2, 3)*	268	3.34
*Range [3, 4)*	5485	68.35
*Range* [4, 5]	2232	17.82
Late retirement (2015)		
*No*	4063	50.64
*Yes*	3961	49.36
Social behavior		
*No*	4299	53.58
*Yes*	3725	46.42
Health insurance		
*Insurance beneficiary (Yes)*	7596	94.67
*Insurance beneficiary (No)*	428	5.33
Gender		
*1. male*	3368	41.97
*2. female*	4656	58.03
Age		
55–65	2255	28.10
66–75	4017	50.07
> 75	1752	21.83
Education		
*Less than lower secondary*	7427	92.64
*Upper secondary & vocational training*	486	6.06
*Tertiary*	104	1.30
Marriage		
*Married*	6372	79.05
*Partnered*	272	3.39
*Separated*	11	0.14
*Divorced*	25	0.31
*Widowed*	1334	16.63
*Never married*	39	0.49
Residence place		
*Central of City/Town*	1316	16.40
*Rural Integration Zone*	445	5.55
*Rural*	6240	77.77
*Special Zone*	23	0.29

*Notes*: The descriptive statistics are based on the observations for which all above variables (including dependent/independent/covariates) have non-missing values

### Analytical strategy

#### Baseline model

The chorological design is important for identifying effects of late retirement on the difficulty in physical functioning and problems of cognitive status. In this study, we use the later retirement (year 2015) as the independent variable, and the difficulty in physical functioning (year 2018) and problems of cognitive status (year 2018) as dependent variables. Such design can to some extent help clarify the effect proposed by prior studies [55]. All other control variables are of year 2018. Thus, the regression model is shown as below.

$$ {\displaystyle \begin{array}{l} Difficulty\ in\ physical\ functioning/ problems\ of\ cognitive\ status\kern0.5em \left( year\ 2018\right)={\beta}_0+{\beta}_1\kern0.5em Late\kern0.5em retirement\ \left( year\ 2015\right)\\ {}+{\beta}_2\kern0.5em Social\ behavior+{\beta}_3\kern0.5em Health\ in surance+{\beta}_4\kern0.5em Age+{\beta}_5\kern0.5em Gender+{\beta}_6\kern0.5em Education+{\beta}_7\kern0.5em Marriage+{\beta}_8\kern0.5em \\ {}\operatorname{Re} sidence\ place+\varepsilon \end{array}} $$

#### Self-selection bias concern: Heckman two-stage regression for robustness check

There might still be some concerns of self-selection bias resulting from the use of non-randomly selected sample, which can bring specification problem [56]. In the context of this study, old-aged adults with better predetermined health status can be more readily to choose late retirement. Prior studies show that poor health can result in the intention of an early exit from the labor market [57]. In this case, the old-aged adults who choose to be late retirees can intrinsically imply the better predetermined health status than those who are not late retirees. As such, Heckman (2013) suggests a two-stage procedure to adjust for the selection bias [56]. In this study, we first regress “*status of working (2015)”* on “*self-reported health (2015)*” and “*health care services (2015)”* using the logit model in the first stage, which predicts the influence of predetermined health on the probability of engagement in work. Specifically, it is shown as below.

$$ {\displaystyle \begin{array}{l} Probability\left[ Status\ of\ working\ \left( year\ 2015\right)=1\right]={\beta}_0+{\beta}_1\kern0.5em Self- reported\ health(2015)+{\beta}_2\kern0.5em Health\ care\ services\ \\ {}\left( year\ 2015\right)+{\beta}_3\kern0.5em Age\ (2015)+{\beta}_4\kern0.5em Gender(2015)+\varepsilon \left[\mathrm{the}\ {1}^{\mathrm{st}}\mathrm{stage}\ \mathrm{regression}\right]\end{array}} $$

Where the variable “*status of working (2015)”* is a binary indicator, and its value equals to 1 if the respondent reports he/she is working in the most recent 1 year (otherwise equals 0). The variable “*health care services (2015)”* indicates the types of health care services the respondent has taken in the last 2 years (i.e., year 2013–2014). Health care services include following items -- “physical examination, routine blood test, routine urine test, liver function test, kidney function test, lipids profile test, blood glucose test, surgical, internal medicine, electrocardiogram, B-type ultrasonic, chest fluoroscopy, ophthalmology and otorhinolaryngology, andrology or gynecology”. The self-reported health is ranged with a 5-point scale from poor to excellent. Through the 1st stage regression, we obtain the Inverse Mills Ratio (IMR), which is used for the adjustment of the potential self-selection bias in the 2nd stage regression. All other control variables are of year 2018. The 2nd stage regression is shown as following.

$$ Difficulty\ in\ physical\ functioning/ problems\ of\ cognitive\ status\ \left( year\  2018\right)={\beta}_0+{\beta}_1\  Inverse\ Mills\ Ratio\ \left( obtained\ from\ the\ {1}^{st}\  stage\ regression\right)+{\beta}_2\  Late\ retirement\ \left( year\  2015\right)+{\beta}_3\  Social\ behavior+{\beta}_4\  Health\ in surance+{\beta}_5\  Age+{\beta}_6\  Gender+{\beta}_7\  Education+{\beta}_8\  Marriage+{\beta}_9\  Residence\ place+\varepsilon\ \left[\mathrm{the}\ {2}^{\mathrm{nd}}\ \mathrm{stage}\ \mathrm{regression}\right] $$

#### Further robustness check: considering the population with past late retirement experience but no longer being late retirees in year 2015

In the above empirical examinations, we only consider the impact of late retirement status of year 2015 (and the time interval since past 1 year) on the difficulty in physical functioning and problems of cognitive status. However, there can be some older adults who have past late retirement experience but are no longer late retirees in the year 2015, and this group of older adults are not taken as late retirees of the year 2015 in this above analysis. In order to further check for robustness, we also conduct two respective regression analyses. The first one is conducted by including this group of older adults in late retirees of the year 2015, and re-examine the influence of late retirement status on the difficulty in physical functioning and problems of cognitive status in this section. The other one is to exclude this group of older adults from the regression. To realize this check, we need to identify the group of older adults with past late retirement experience but no longer being late retirees in year 2015 (and the most recent 1 year). In the survey, respondents are inquired about the time that they stop working. Older adults who stop working at aged > 60 for males (> 55 for females) and do not work in year 2015 (and the most recent 1 year) are categorized as in this group.

## Empirical results

### Results of baseline model

Results of Table 3 provide a preliminary comparison of difficulty in physical functioning and problems of cognitive status between the groups of late retirees and non-late retirees. Results show that there is significant difference between these two groups of older adults. More specifically, the non-late retirees have higher mean value of difficulty in physical functioning than late retirees (*Mean*_*non-late retirees*_ = 1.937, and *Mean*_*late retirees*_ = 1.567, *p-value* < 0.01); the non-late retirees have higher mean value of problems of cognitive status than late retirees (*Mean*_*non-late retirees*_ = 3.632, and *Mean*_*late retirees*_ = 3.559, *p-value* < 0.01).

**Table 3:** The comparison of difficulty in physical functioning and cognitive status between groups of late retirees and non-late retirees

	*Obs.*	*Mean*	*S. D.*	*95% CI*	*T-value of the diff.*	*p-value*
**Sample 1: difficulty in physical functioning (2018)**						
*Late retirees (2015)*	4791	1.567	0.555	[1.916, 1.956]	27.863	0.000
*Non-late retirees (2015)*	5236	1.937	0.748	[1.552, 1.583]		
**Sample 2: problems of cognitive status (2018)**						
*Late retirees (2015)*	4168	3.559	0.628	[3.540, 3.578]	5.321	0.000
*Non-late retirees (2015)*	4499	3.632	0.649	[3.614, 3.651]		

*Notes*: The comparison between late retirees and non-late retiree is based the observations for which the independent variable (i.e., late retirement status) has non-missing values. Thus, the number of observations with non-missing value shown here is not exactly as that shown in Table 2 which has excluded observations with missing value in covariates

Table 4 demonstrates the results of baseline models. Results show that older adults who become a late retiree in the year of 2015 have experienced less difficulty in physical functioning (coefficient = − 0.343, 95% CI [− 0.373, − 0.313], *p* < 0.01) and less problems of cognitive status (coefficient = − 0.089, 95% CI [− 0.121, − 0.058], *p* < 0.01) in the year of 2018.

**Table 4:** The influence of late retirement on difficulty in physical functioning and problems of cognitive status

	Dependent variables					
	*Difficulty in physical functioning (2018)*			*Problems of cognitive status (2018)*		
	Coef.	S.E.	95% CI	Coef.	S.E.	95% CI
Late retirement status of 2015	− 0.343 ^**^	0.015	[− 0.373, − 0.313]	− 0.089 ^**^	0.016	[− 0.121, − 0.058]
Social behavior	− 0.182 ^**^	0.013	[− 0.207, − 0.156]	− 0.092 ^**^	0.014	[− 0.120, − 0.064]
Health insurance	− 0.011	0.028	[− 0.066, 0.044]	0.085 ^**^	0.029	[0.029, 0.141]
Gender						
*Male*	REF.			REF.		
*Female*	0.252 ^**^	0.014	[0.225, 0.280]	0.032 ^*^	0.015	[0.003, 0.062]
Age	0.022 ^**^	0.001	[0.020, 0.024]	0.013 ^**^	0.001	[0.011, 0.016]
Education						
*Less than lower secondary*	REF.			REF.		
*Upper, secondary & vocational training*	−0.161 ^**^	0.023	[− 0.206, − 0.117]	− 0.094 ^**^	0.026	[− 0.146, − 0.043]
*Tertiary*	− 0.229 ^**^	0.042	[− 0.311, − 0.147]	− 0.108 ^*^	0.046	[− 0.199, − 0.017]
Marriage						
*Married*	REF.			REF.		
*Partnered*	− 0.046	0.031	[− 0.107, 0.014]	− 0.031	0.037	[− 0.103, 0.042]
*Separated*	−0.074	0.164	[− 0.395, 0.246]	− 0.216	0.190	[− 0.588, 0.156]
*Divorced*	0.127	0.091	[− 0.051, 0.306]	0.079	0.121	[−0.158, 0.316]
*Widowed*	0.025	0.019	[−0.013, 0.062]	−0.019	0.020	[−0.059, 0.021]
*Never married*	0.141	0.072	[0.000, 0.281]	0.109	0.099	[−0.086, 0.303]
Residence place						
*Central city/town*	REF.			REF.		
*Urban-rural integration zone*	0.089 ^**^	0.029	[0.032, 0.145]	0.042	0.031	[−0.019, 0.103]
*Rural*	0.259 ^**^	0.019	[0.223, 0.296]	0.214 ^**^	0.020	[0.176, 0.253]
*Special zone*	0.089	0.111	[−0.129, 0.306]	−0.036	0.103	[−0.237, 0.166]
Intercept	0.132	0.089	[−0.042, 0.306]	2.480 ^**^	0.094	[2.296, 2.665]
Observations	9126			8024		
F-statistics	157.76			34.96		
[*P*-value]	[0.000]			[0.000]		

Notes: ^*^p < 0.05, ^**^p < 0.01

### Robustness check with Heckman two-stage procedure to address potential self-selection bias

Empirical results (Table 5) show the results of robustness check using Heckman two-stage regression, which again confirms the influence of late retirement on difficulty in physical functioning (coefficient = − 0.353, 95% CI [− 0.399, − 0.308], *p* < 0.01) and problems of cognitive status (coefficient = − 0.069, 95% CI [− 0.119, − 0.018], *p* < 0.01) of old-aged adults after the adjustment of potential self-selection bias.

**Table 5:** The influence of late retirement on difficulty in physical functioning and problems of cognitive status (Heckman two-stage regression)

	Dependent variables					
	*Difficulty in physical functioning (2018)*			*Problems of cognitive status (2018)*		
	Coef.	S.E.	95% CI	Coef.	S.E.	95% CI
Late retirement status of 2015	−0.353^**^	0.023	[−0.399, − 0.308]	−0.069 ^**^	0.026	[−0.119, − 0.018]
Social behavior	− 0.130 ^**^	0.020	[− 0.169, − 0.091]	− 0.084 ^**^	0.023	[− 0.129, − 0.039]
Health insurance	− 0.045	0.042	[− 0.127, 0.037]	0.121 ^**^	0.043	[0.036, 0.205]
Gender						
*Male*	REF.			REF.		
*Female*	−0.317 ^**^	0.060	[−0.435, − 0.199]	−0.207 ^**^	0.059	[−0.323, − 0.091]
Age	− 0.047 ^**^	0.007	[− 0.060, − 0.033]	− 0.011	0.007	[− 0.025, 0.002]
Education						
*Less than lower secondary*	REF.			REF.		
*Upper, secondary & vocational training*	−0.194 ^**^	0.039	[− 0.271, − 0.116]	−0.077	0.049	[−0.174, 0.020]
*Tertiary*	−0.284 ^**^	0.084	[− 0.448, − 0.120]	−0.095	0.111	[−0.313, 0.123]
Marriage						
*Married*	REF.			REF.		
*Partnered*	−0.030	0.046	[−0.120, 0.061]	− 0.015	0.059	[−0.131, 0.100]
*Separated*	0.205	0.229	[−0.244, 0.654]	−0.036	0.185	[−0.399, 0.327]
*Divorced*	−0.030	0.128	[−0.282, 0.221]	0.046	0.177	[−0.301, 0.392]
*Widowed*	0.011	0.031	[−0.049, 0.071]	− 0.002	0.034	[−0.068, 0.064]
*Never married*	0.017	0.099	[−0.177, 0.211]	0.002	0.156	[−0.305, 0.308]
Residence						
*Central city/town*	REF.			REF.		
*Urban-rural integration zone*	0.137 ^**^	0.049	[0.041, 0.234]	0.025	0.057	[−0.086, 0.136]
*Rural*	0.269 ^**^	0.030	[0.211, 0.328]	0.161 ^**^	0.034	[0.094, 0.228]
*Special zone*	0.096	0.208	[−0.311, 0.503]	−0.086	0.184	[−0.446, 0.274]
Inverse mills ratio	1.061 ^**^	0.104	[0.856, 1.265]	0.384 ^**^	0.102	[0.184, 0.584]
Intercept	4.353 ^**^	0.435	[3.500, 5.207]	4.010 ^**^	0.428	[3.172, 4.849]
Observations	3757			3223		
F-statistics	69.27			10.44		
[*P*-value]	[0.000]			[0.000]		

*Notes*: The Heckman two-stage regression (1st stage) is conducted by regressing late retirement (2015) on self-reported health (2015), health care services (2015), age and gender, according to which the Inverse Mills Ratio (IMR) is obtained and applied in the 2nd stage regression.

^*^p < 0.05, ^**^p < 0.01

### Robustness check by considering the population with past late retirement experience but have stopped working in year 2015 and thus no longer being late retirees (2015)

In the above analysis, older adults who have past late retirement experience but have stopped working in year 2015 are not taken as belonging to the late retirement status (2015). In order to check for robustness, we also include this group of older adults as late retirees (2015) in the regression analysis. Results of Table 6 (Panel A) display that the above results remain significant. Specifically, the influence of late retirement on difficulty in physical functioning (coefficient = − 0.210, 95% CI [− 0.241, − 0.180], *p* < 0.01) and problems of cognitive status (coefficient = − 0.048, 95% CI [− 0.079, − 0.017], *p* < 0.01).

**Table 6:** Robustness check: considering the population with past late retirement experience but having stopped working in year 2015

	Dependent variable (of year 2018)											
	Panel A						Panel B					
	*Difficulty in physical functioning*			*Problems of cognitive status*			*Difficulty in physical functioning*			*Problems of cognitive status*		
	Coef.	S.E.	95% CI	Coef.	S.E.	95% CI	Coef.	S.E.	95% CI	Coef.	S.E.	95% CI
Late retirement status of 2015	−0.210 ^**^	0.016	[−0.241, −0.180]	−0.048 ^**^	0.016	[−0.079, − 0.017]	−0.331 ^**^	0.017	[−0.365, − 0.297]	−0.080 ^**^	0.018	[−0.115, − 0.045]
Social behavior	− 0.181 ^**^	0.013	[− 0.206, − 0.155]	−0.091 ^**^	0.014	[−0.119, − 0.063]	−0.152 ^**^	0.014	[−0.180, − 0.124]	−0.089 ^**^	0.016	[−0.120, − 0.058]
Health insurance	0.012	0.029	[−0.069, 0.045]	0.085 ^**^	0.029	[0.028, 0.141]	0.000	0.031	[−0.060, 0.061]	0.093 ^**^	0.032	[0.031, 0.155]
Age	0.028 ^**^	0.001	[0.026, 0.031]	0.041 ^**^	0.015	[0.012, 0.071]	0.021 ^**^	0.001	[0.018, 0.023]	0.033 ^*^	0.017	[0.001, 0.066]
*Male*	REF.			REF.			REF.			REF.		
*Female*	0.285 ^**^	0.014	[0.258, 0.312]	0.015 ^**^	0.001	[0.013, 0.017]	0.238 ^**^	0.015	[0.208, 0.268]	0.013 ^**^	0.001	[0.010, 0.016]
Education												
*Less than lower secondary*	REF.			REF.			REF.			REF.		
*Upper, secondary & vocational training*	−0.151 ^**^	0.023	[−0.191, −0.107]	−0.091 ^**^	0.026	[−0.142, − 0.039]	−0.162 ^**^	0.024	[−0.209, − 0.116]	−0.087 ^**^	0.028	[−0.142, − 0.032]
*Tertiary*	− 0.197 ^**^	0.043	[− 0.281, − 0.114]	−0.100 ^*^	0.047	[−0.192, − 0.009]	−0.254 ^**^	0.048	[−0.347, − 0.160]	−0.099	0.057	[−0.210, 0.013]
Marriage												
*Married*	REF.			REF.			REF.			REF.		
*Partnered*	−0.049	0.031	[−0.110, 0.011]	− 0.033	0.037	[− 0.105, 0.040]	−0.014	0.033	[−0.079, 0.050]	− 0.025	0.039	[− 0.102, 0.051]
*Separated*	−0.041	0.176	[−0.386, 0.304]	− 0.201	0.186	[− 0.566, 0.163]	−0.111	0.138	[−0.381, 0.159]	− 0.029	0.161	[− 0.343, 0.286]
*Divorced*	0.179	0.094	[−0.006, 0.364]	0.093	0.122	[−0.146, 0.333]	0.060	0.106	[−0.147, 0.268]	0.064	0.142	[−0.214, 0.342]
*Widowed*	0.045	0.019	[0.007, 0.083]	−0.014	0.020	[−0.054, 0.026]	0.034	0.022	[−0.009, 0.078]	−0.010	0.024	[−0.058, 0.037]
*Never married*	0.170	0.074	[0.024, 0.316]	0.122	0.100	[−0.075, 0.318]	0.147	0.078	[−0.006, 0.300]	0.057	0.109	[−0.157, 0.271]
Residence place												
*Central city/town*	REF.			REF.			REF.			REF.		
*Urban-rural integration zone*	0.085 ^**^	0.029	[0.028, 0.141]	0.040	0.031	[−0.021, 0.101]	0.105 ^**^	0.031	[0.043, 0.166]	0.056	0.034	[−0.011, 0.123]
*Rural*	0.207 ^**^	0.019	[0.171, 0.243]	0.198 ^**^	0.020	[0.159, 0.236]	0.246 ^**^	0.021	[0.205, 0.287]	0.196 ^**^	0.022	[0.153, 0.239]
*Special zone*	0.017	0.111	[−0.201, 0.235]	−0.054	0.103	[−0.257, 0.148]	0.086	0.116	[−0.141, 0.314]	−0.044	0.103	[−0.247, 0.158]
Intercept	0.331 ^**^	0.086	[−0.201,0.235]	2.354 ^**^	0.091	[2.176, 2.533]	0.205 ^*^	0.103	[0.004,0.406]	2.502 ^**^	0.108	[2.290, 2.715]
Observations	9118			8016			7345			6455		
F-statistics	133.48	[0.00]		33.76	[0.00]		96.75	[0.00]		22.08		
[*P*-value]	[0.000]			[0.000]			[0.000]			[0.000]		

Dependent variables are of year 2018. Panel A (and B) is conducted by including (and excluding) the population with past late retirement experience but having stopped working in year 2015 into (or from) the group of late retirees (2015))

*Notes*: ^*^p < 0.05, ^**^p < 0.01

Besides, we also try to exclude the population with past late retirement experience but have stopped working in year 2015 from the sample. Results of Table 6 (Panel B) demonstrate that the above results remain significant. Specifically, the influence of late retirement on difficulty in physical functioning (coefficient = − 0.331, 95% CI [− 0.365, − 0.297], *p* < 0.01) and problems of cognitive status (coefficient = − 0.080, 95% CI [− 0.115, − 0.045], *p* < 0.01).

## Discussion

### General discussion

Prior studies about delayed retirement mainly focus on the fiscal pressure of pension schemes and labor supply in the labor market in the context of population aging. The main concerns of policymakers surround the effect of delayed retirement on the attenuation of labor shortage and the alleviation of heavy pension burden. There lacks sufficient treatment regarding the health outcomes of late retirees. This study turns attention to this issue and fortunately the potential beneficial effect of late retirement on health of older adults is found.

This study reveals health implications of late retirement, and suggests that late retirement can alleviate the difficulty in physical functioning and problems of cognitive status of older adults. While recognizing the association between health and “late retirement” could not be directly mirrored from that between health and retirement, this finding is consistent with previous indirect empirical evidence regarding the association between an early exit from the labor market and a significant negative impact on cognitive status of older adults in the US, UK, and other European countries [27]. Previous indirect evidence also demonstrates that worse mental health status is closely associated with the continual detachment from work status during the retirement transition period [58]. The trajectory of retirement age and mental health disorder also displays that older adults who exit the labor market earlier than their counterparts experience poorer mental health, and the continual engagement in employment among older adults is suggested in order to lower down relevant risks [59]. Late retirement can be associated with reduced negative effects of aging on physical function and cognitive status, as it helps maintain social and physical activities with moderate intensity [26]. Thus, the modest work engagement in late life can alleviate some common physical and cognitive symptoms, such as dementia [35] and depression [47].

### Practical implications

The period of retirement transition is critical for the health of older adults. There have been some studies showing that the sudden change of working status from full-load work to detachment from work may have negative impact on the health of older adults, such as depressive symptoms, physical inactivity, reduced opportunities of communication or social contact with others, and the inability to reach personal need fulfillment for identity [60]. Thus, from the perspective of individual health of older adults, the gradual transition of retirement period can be beneficial. In this study, it is shown that the engagement in careers at old-age might be beneficial for both physical health and cognitive status of older adults. For policymakers, it is helpful to advocate the voluntary late retirement of older adults, and introduce policies to encourage companies to employ older adults. The incentive measure is more helpful to build a friendly and humanized environment of late retirement than merely lifting the legal retirement age in order to alleviate the fiscal pressure to pay pension or address the labor shortage problem.

### Limitations

This study also has some limitations. First, the results of this study may be contextually contingent. Subjected to the availability of data, we cannot differentiate the effect according to different job types engaged in by older adults in their late careers. However, different job types might lead to very different results. For some jobs with high physical demands, the engagement in these jobs in late career might not significantly benefit cognitive status of older adults. Second, we are not able to differentiate the intensity of work engagement of older adults in late careers with the current data. However, prior studies show that modest work engagement in late career is beneficial to health whereas overwork appears not [61]. Third, there might be difference in delayed retirement policies across countries, which can induce different incentives of participation in late careers and different disincentives of an early exit from the labor market. Thus, the health implications cannot be exactly the same under different backgrounds of delayed retirement policies. An in-depth investigation on the health implications of different delayed retirement policies is in need for future research.

## Conclusion

This study intended to enrich research on late retirement by examining the relationships among late retirement, difficulty in physical functioning, and problems of cognitive status. Late retirement was found to be associated with better physical functioning and cognitive status. A series of robustness checks further confirmed these findings. Incentives for older adults to delay retirement need to be taken into account in national policy formulation.

## Data Availability

The data used in this study are open source and publicly available. The data are available upon reasonable request via online application to the China Health and Retirement Longitudinal Study (CHARLS) research program.

## Abbreviations

OECD: Organization for Economic Co-operation and Development
CHARLS: China Health and Retirement Longitudinal Study
